# Functional and Anatomical Outcomes of Pars Plana Vitrectomy for Lamellar Macular Hole: Long-Term Follow-Up

**DOI:** 10.3390/diagnostics15010027

**Published:** 2024-12-26

**Authors:** Fabrizio Giansanti, Cristina Nicolosi, Giuseppe Ruben Barbera, Giulio Vicini, Flavia Lucarelli, Edoardo Traniello Gradassi, Vittoria Murro, Gianni Virgili, Daniela Bacherini

**Affiliations:** 1 Eye Clinic, Neuromuscular and Sense Organs Department, Careggi University Hospital, Largo Brambilla 3, 50134 Florence, Italy; fabrizio.giansanti@unifi.it (F.G.); flavia.lucarelli@unifi.it (F.L.); edoardo.traniellogradassi@unifi.it (E.T.G.); vittoria.murro@unifi.it (V.M.); gianni.virgili@unifi.it (G.V.); daniela.bacherini@gmail.com (D.B.); 2 Department of Neurosciences, Psychology, Drug Research and Child Health, University of Florence, 50134 Florence, Italy; giulio.vicini@gmail.com; 3 Azienda USL Toscana Nord Ovest, 56121 Pisa, Italy

**Keywords:** lamellar macular hole, pars plana vitrectomy, functional outcomes, anatomical outcomes, macular surgery

## Abstract

Background: To investigate functional and anatomical outcomes after pars plana vitrectomy (PPV) for lamellar macular hole (LMH) with a long-term follow-up. Methods: An interventional study on 14 patients (16 eyes) with LMH was conducted. The inclusion criteria included a minimum 36-month follow-up after PPV. The preoperative and postoperative best-corrected visual acuity (BCVA) and spectral domain optical coherence tomography parameters were examined. Results: Preoperatively, the mean BCVA was 0.46 ± 0.22 LogMAR. Epiretinal proliferation (ERP) was visible in 81.25% of eyes. Outer retinal disruption was present in 31.25% of LMH cases. The average central foveal thickness (CFT) measured 183.68 ± 61.73 microns. The mean BCVA improved at each follow-up time point: it was 0.24 ± 0.16 LogMAR at 1 month, 0.18 ± 0.15 LogMAR at 6 months, and 0.09 ± 0.11 LogMAR at the last follow-up. There was a statistically significant improvement between BCVA at 1 month and BCVA at 6 months and between BCVA at 6 months and BCVA at the last follow-up (*p* < 0.001). BCVA improved in all eyes, with 87.5% achieving at least 0.3 LogMAR improvement. The mean CFT at the 1-month follow-up was 211.45 ± 43.55 microns, increased to 248.81 ± 48.51 microns at 6 months, and further increased to 278.37 ± 45.50 microns at the last follow-up. Foveal contour restoration was achieved in all eyes, and those with preoperative ellipsoid zone alterations demonstrated a complete repair of the external retinal layers. No intra or postoperative complications were recorded. Conclusions: In our series, PPV had a high success rate and was associated with a substantial functional improvement in LMH treatment, and this result was maintained and kept increasing until the last follow-up. Long-term follow-up is crucial for a comprehensive evaluation of the healing process and to assess the benefits of the surgical intervention.

## 1. Introduction

Lamellar macular hole (LMH) is a macular disorder characterized by a partial-thickness foveal defect with irregular edges. In 2016, Govetto et al. introduced a classification system for LMHs, which distinguishes two subtypes based on distinct pathogenetic and clinical features: degenerative LMH and tractional LMH [1]. The degenerative subtype is a partial-thickness defect in the inner fovea with a foveal cavity with undermined edges, associated with the presence of lamellar hole-associated epiretinal proliferation, frequent disruption of the outer retina, and, in some cases, the appearance of a central bump of spared foveal tissue. The tractional subtype, on the other hand, is characterized by foveoschisis at the level of the Henle’s fiber layer, the presence of a tractional epiretinal macular membrane (ERM), intact photoreceptors, and microcystic macular edema in the inner nuclear layer (INL). Recently, in the hopes of facilitating future research, an international panel of vitreoretinal experts published a Spectral Domain OCT (SD-OCT)-based consensus definition for LMH [2]. In this new classification, tractional LMH were renamed as ERM foveoschisis, whereas degenerative LMH were renamed as LMH. The group of retinal experts agreed that the definition of LMH should be based on three mandatory criteria: (a) irregular foveal contour; (b) foveal cavity with undermined edges; and the (c) presence of at least one other sign evoking a loss of foveal tissue (pseudo-operculum, thinning of the fovea at its centre, or around). Additional diagnostic criteria can include the following: (a) epiretinal proliferation (ERP); (b) foveal bump; and (c) ellipsoid line disruption. Most individuals affected by LMH complain of slight central visual impairment and/or metamorphopsia, although this condition remains unchanged on OCT over an extended period. A small subset of patients with LMH may experience more pronounced visual decline and/or exhibit an anatomical worsening on OCT scans [3,4]. Currently, there is no consensus about the management of LMH, the choice of observation versus treatment in stable cases, or the timing of treatment of LMH, due to the lack of clear guidelines from existing research. Therefore, we decided to conduct the present study on the long-term outcome of patients affected by LMH that underwent surgical treatment, followed by an ophthalmological examination and OCT scans for a minimum period of 36 months, in order to evaluate the utility of pars plana vitrectomy (PPV) in the long-term anatomical restoration and functional recovery.

## 2. Material and Methods

We conducted an interventional study on patients affected by LMH who underwent PPV and ERP and internal limiting membrane (ILM) peeling at a single center (Eye Clinic, Careggi University Hospital, Florence, Italy), and were followed up with a comprehensive ophthalmological examination and OCT scans for a minimum period of 36 months. The study was approved by the Institutional Review Board and followed the principles of the Declaration of Helsinki. Informed written consent was obtained from all participants. The clinical records and OCT images of 36 eyes of 30 patients who underwent vitrectomy for LMH, performed by an expert vitreoretinal surgeon (F.G.) between 1 January 2015 and 1 January 2020, were examined and reviewed by two ophthalmologists, expert in retinal pathologies (C.N. and D.B.).

### 2.1. Inclusion and Exclusion Criteria

**Figure 1:**
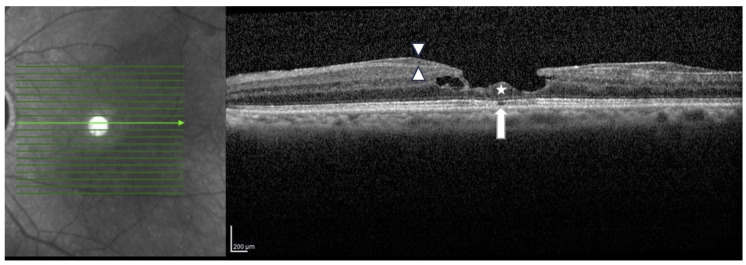
Lamellar Macular Hole Spectral Domain Optical Coherence Tomography (SD-OCT) imaging. The OCT scan reveals a characteristic lamellar macular hole, featuring an irregular foveal contour, a foveal cavity with undermined edges, a foveal thinning and the presence of epiretinal proliferation (heads of arrows) and foveal bump (star). Additionally, disruption in the ellipsoid line and the external limiting membrane (arrow) can be observed.

The inclusion criteria for the study were the diagnosis of LMH and a surgical intervention with PPV, ERP, and ILM peeling performed at least 36 months before, availability of preoperative and postoperative (at 1 and 6 months) OCT scans, availability of preoperative best-corrected visual acuity (BCVA), and postoperative BCVA data at 1 and 6 months after surgery. All patients who did not meet the inclusion criteria were excluded. The diagnosis of LMH was established according to the OCT-based consensus criteria [2] (Figure 1).

Exclusion criteria for the study were as follows: media opacities as cataract, not allowing proper preoperative OCT imaging, advanced age-related macular degeneration, glaucoma, diabetic retinopathy, high myopia (>6 D) or myopic choroidal neovascularization, vascular retinal diseases, macular telangiectasias, uveitis and intraocular infections, retinal dystrophies, trauma and any previous intraocular surgery, as well as amblyopia, keratoconus, corneal dystrophies, optic neuropathy and central serous chorioretinopathy.

Due to the availability of comprehensive data and imaging, clinical records and OCT images from 36 eyes of 30 patients were reviewed. However, only 14 patients (16 eyes) met all inclusion criteria and were ultimately included in the interventional study.

### 2.2. Surgery

All patients were symptomatic and reported a worsening of visual acuity and/or metamorphopsia before surgery. We indicated surgery for patients with LMH who experienced worsening symptoms, such as distortion or decreased visual acuity, or had documented changes on OCT. The presence of an interrupted ellipsoid zone (EZ) or external limiting membrane band in the foveal region was considered a sign of photoreceptor impairment and disruption.

All surgical operations were performed under peribulbar anesthesia. For all patients, standard cataract surgery with intraocular lens (IOL) implantation was combined with vitrectomy. All eyes underwent a standard sutureless 25-gauge three-port pars-plana vitrectomy with a wide-angle non-contact viewing system (Resight^®^; Carl Zeiss Meditec AG, Jena, Germany) using the Constellation Vision System (Alcon Laboratories Inc., Fort Worth, TX, USA). To stain and peel the ILM and ERP when present, 0.18% Trypan Blue and 0.03% Blulife were used. ILM was peeled up to a diameter of 2 papillary widths. A complete vitrectomy was performed, and during the procedure, triamcinolone solution was used to stain the vitreous. At the end of the surgery, fluid–air–gas exchange was performed using an isoexpansile mixture of sulphur hexafluoride gas (SF6-20%). Postoperatively, patients were instructed to maintain a face-down position for a period of 3 to 5 days.

### 2.3. Patients Examination and Data Collection

We collected preoperative and post-operative clinical and OCT data at 1 and 6 months of follow-up after surgery. BCVA was initially reported in decimal and then converted to logarithm of the minimal angle of resolution (logMAR) values for statistical analysis. The recorded preoperative and postoperative OCT scans were captured using the following machines: RS-3000, Nidek (Gamagori, Japan); Spectralis HRA + OCT, Heidelberg Engineering (Heidelberg, Germany); RTVue, Optovue (Freemont, CA, USA).

The following preoperative parameters were collected: patients’ age and gender, BCVA, Central Foveal Thickness (CFT), and subjective metamorphopsia tested with Amsler grid. The CFT was determined by means of automated measurement by the OCT software. We also performed a qualitative OCT analysis, including the evaluation of EZ disruption and the presence of ERP. ERP was defined according to Pang et al. [4] as a thick and uniform isoreflective material located above the ILM and covered by a thin hyperreflective line. There should be no signs of wrinkling in the underlying retina and no presence of hyporeflective spaces between ERP and the ILM.

In patients that met the inclusion criteria and were eligible for the study, a comprehensive ophthalmological examination and SD-OCT scans were repeated to obtain a long-term follow-up evaluation. For the last examination of enrolled patients, we used a SD-OCT (Heidelberg, Germany), and a minimum of two types of SD-OCT scan patterns were used. These included 19 B-scans taken with a pattern size of 20 × 15° with each B-scan spaced 242 µm apart, as well as a single high-definition horizontal B-scan at 30°. Some of the eyes also underwent volume scan (posterior pole) over a macular area of 30 × 25° with 61 B-scans, each spaced 122 µm apart. At the last follow-up, we also tested the presence of metamorphopsia with Amsler grid test and we asked patients about the subjective distortion sensation. The following quantitative postoperative parameters (at the 1-month follow-up, 6 months follow-up and at the last follow-up) were collected: BCVA and CFT. We also performed a qualitative analysis of post-operative OCT parameters, as the recurrence of ERP and the EZ disruption evaluation. We analyzed the occurrence of complications after surgery, including macular holes, retinal detachment, and endophthalmitis. No patients were lost to follow-up at any point during the study, ensuring complete data collection across all follow-up visits.

### 2.4. Primary Endpoints and Statistical Analysis

Primary outcomes measured were the improvement in BCVA and the anatomical restoration of foveal contour over a period of at least 36 months since PPV. The secondary outcomes included the evaluation of post-operative CFT, modifications in EZ disruption, subjective modifications in metamorphopsia perception and prevalence of complications. We also investigated the correlation between BCVA and CFT as a secondary outcome.

The statistical analysis was carried out using Stata 18.0 (StataCorp, College Station, TX, USA).

We investigated the change in BCVA and CFT from baseline during the follow-up using linear mixed models, with individuals as random intercept and time as random slope. In particular, we made comparisons between preoperative values and those at the 1-month follow-up, between values at 1 month and 6 months, and between values at 6 months and the last follow-up, to assess short-term impacts, intermediate changes, and long-term outcomes of the intervention. Additionally, we performed correlation analyses between BCVA and CFT at 6 months, and at the last follow-up using Spearman’s rank correlation coefficient. This analysis provided further insight into the relationship between functional and anatomical improvements following surgery. A *p*-value of less than 0.05 was considered statistically significant. The results are presented as mean ± standard deviation.

## 3. Results

### 3.1. Before Surgery

The study included a total of 14 patients and 16 eyes, 4 male individuals (28.57%) and 10 female individuals (71.43%), who underwent surgery at an average age of 76.1 ± 9.9 years. At the time of presentation, all patients were phakic.

Before surgery, the mean BCVA was 0.46 ± 0.22 LogMAR. ERP was found in 13 eyes (81.25%). At the preoperative assessment, 5 out of 16 eyes (31.3%) diagnosed with LMH had EZ disruption. The average CFT was 183.68 ± 61.73 microns. Six patients (37.5%) complained of metamorphopsia on the Amsler test at baseline. Table 1 reports patients baseline demographical, functional, and quantitative and qualitative structural OCT characteristics.

### 3.2. After Surgery

**Figure 2:**
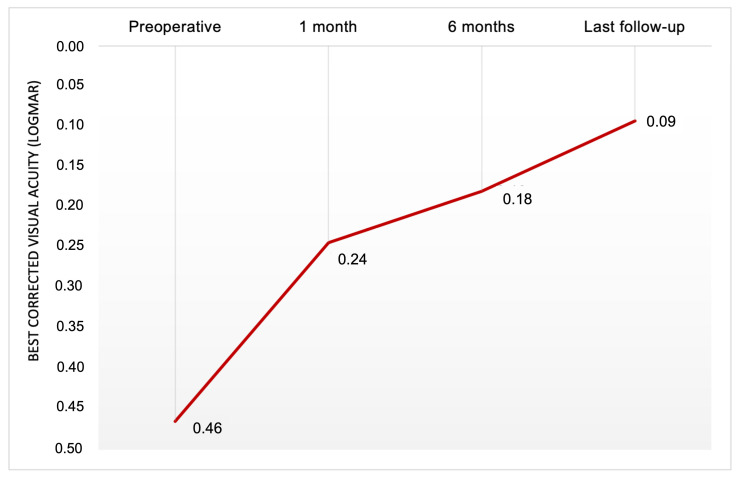
Preoperative and postoperative (at 1 month, at 6 months, at last follow-up) best-corrected visual acuity (BCVA) for patients affected by lamellar macular hole who underwent pars-plana vitrectomy. The mean BCVA improved at each follow-up time point. Significant improvement was observed between preoperative BCVA and BCVA at 1 month (*p* < 0.001). The improvement between BCVA at 1 month and BCVA at 6 months was not statistically significant (*p* = 0.072). There was a significant improvement between BCVA at 6 months and BCVA at the last follow-up (*p* < 0.001).

**Figure 3:**
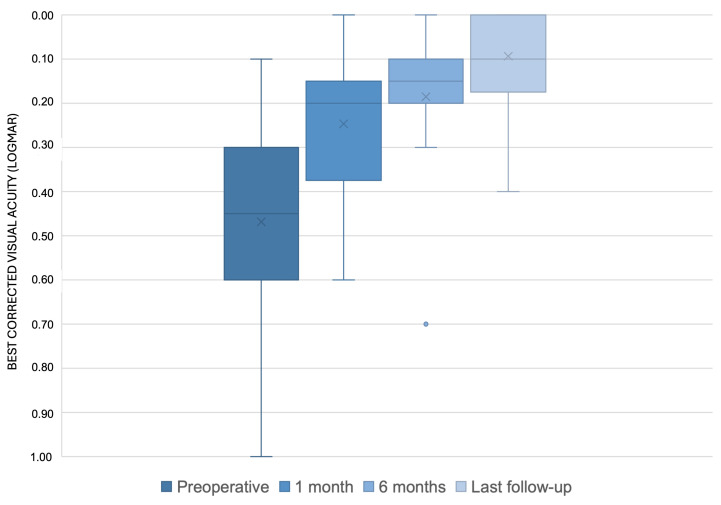
Box-plot of best-corrected visual acuity (BCVA) at the baseline and each follow-up time of the study. The BCVA improved by at least 0.3 LogMAR in 14 eyes (87.5%) when comparing the baseline to the last follow-up.

The follow-up period ranged from a minimum of 36 months to a maximum of 74 months, with an average of 54.9 ± 13.1 months. All 16 eyes showed an improvement in vision after surgery. The mean BCVA improved at each follow-up time point: it was 0.24 ± 0.16 LogMAR at 1 month, 0.18 ± 0.15 LogMAR at 6 months, and 0.09 ± 0.11 LogMAR at the last follow-up, with a range from 0.4 LogMAR to 0.0 LogMAR (Figure 2 and Figure 3). Significant improvement was observed between preoperative BCVA and BCVA at 1 month (*p* < 0.001). The improvement between BCVA at 1 month and BCVA at 6 months was not statistically significant (*p* = 0.072). There was a significant improvement between BCVA at 6 months and BCVA at the last follow-up (*p* < 0.001).

The BCVA improved by at least 0.3 LogMAR in 14 eyes (87.5%) when comparing the baseline to the last follow-up. Of these, 7 eyes (43.8%) showed an improvement of at least 0.4 LogMAR. Only 2 eyes (12.5%) had an increase in BCVA of less than 0.3 LogMAR, but these eyes improved from 0.1 to 0 LogMAR and from 0.2 to 0 LogMAR, respectively, from the baseline to the last follow-up (Figure 2 and Figure 3). At the last follow-up, the Amsler grid test was still positive in four patients; however, these patients reported a subjective reduction in metamorphopsia.

**Figure 4:**
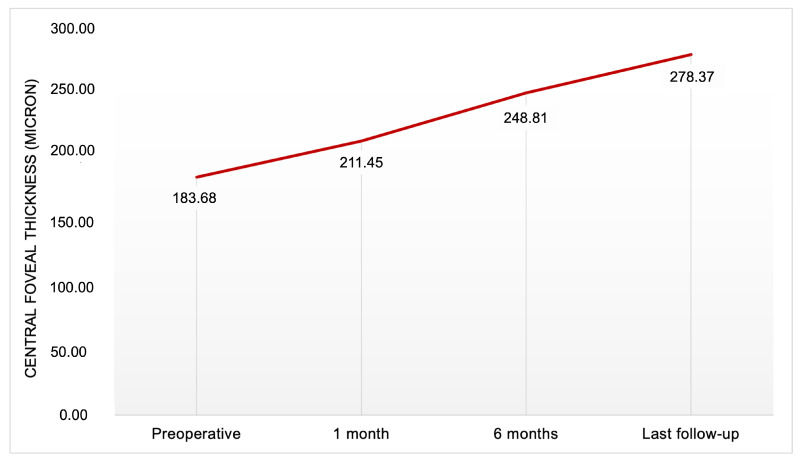
Preoperative and postoperative (at 1 month, at 6 months, at last follow-up) CFT (central foveal thickness) for patient affected by lamellar macular hole who underwent pars-plana vitrectomy. The increase in CFT was statistically significant (*p* < 0.001) at each follow-up time point.

**Figure 5:**
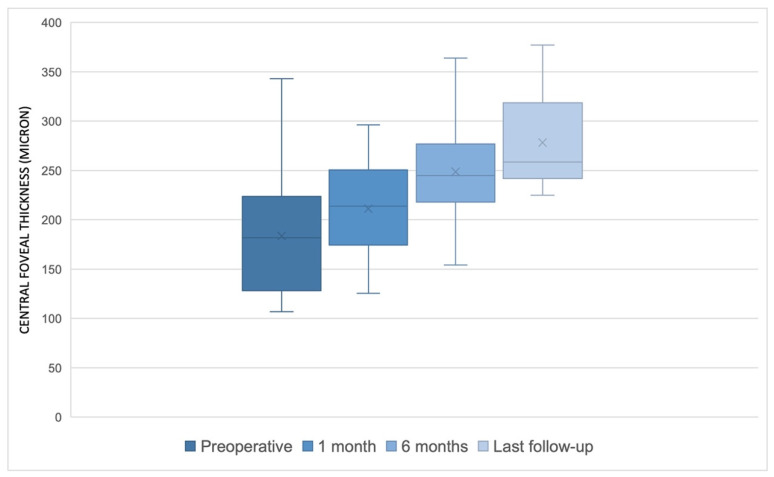
Box-plot of central foveal thickness (CFT) at baseline and each follow-up time of the study.

The mean CFT at the 1-month follow-up was 211.45 ± 43.55 microns, ranging from 124.35 to 298.55 microns. At 6 months follow-up, it was 248.81 ± 48.51 microns, ranging from 154 to 364 microns. At the last follow-up, CFT was 278.37 ± 45.50 microns, with a range of 225 to 377 microns. The increase in CFT was statistically significant (*p* < 0.001) at each follow-up time point: from baseline values to 1 month (increase of 27.7 microns), from 1 month to 6 months (increase of 37.36 microns), and from 6 months to the last follow-up (increase of 29.56 microns) (Figure 4 and Figure 5). Regarding CFT, 3 eyes (18.75%) exhibited an increase at 6 months, which remained stable at the last follow-up. In one eye, CFT decreased at 6 months but showed improvement at the last follow-up. In all other patients, there was an increase in CFT from 6 months to the last follow-up.

At 6 months, we found a moderate positive correlation between the increase in BCVA and the increase in CFT, though it did not reach statistical significance (Spearman R = 0.44, *p* = 0.086). This correlation was further reduced at the last follow-up (Spearman R = 0.011, *p* = 0.689). Preoperative and Postoperative BCVA and CFT in eyes affected by lamellar macular hole who underwent pars plana vitrectomy are shown in Table 2 and Table 3, respectively.

**Figure 6:**
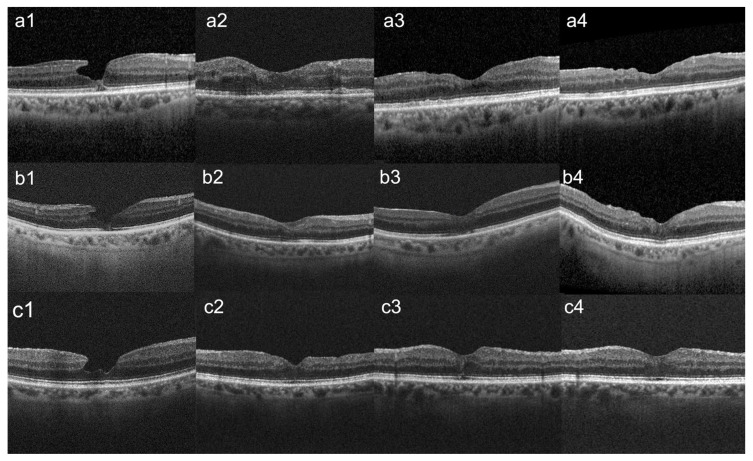
Preoperative and postoperative Optical Coherence Tomography appearance of lamellar macular hole in three patients. (**a1**–**c1**): Preoperative appearance. (**a2**–**c2**): Postoperative appearance at the 1-month follow-up. (**a3**–**c3**): Postoperative appearance at 6 months follow-up. (**a4**–**c4**): Postoperative appearance at the last follow-up (minimum 36-month after pars-plana vitrectomy).

We also performed a qualitative OCT analysis, and we found that at the last follow-up, all 16 eyes (100%) had restoration of the foveal contour and the disappearance of intraretinal cavitation after surgery. In total, 5 out 5 eyes with preoperative EZ alteration had complete repair of the defect (Figure 6). Furthermore, the qualitative assessment of OCT images revealed the absence of ERP recurrence after surgery. We did not observe macular edema at the different follow-up examinations. No complications were recorded after surgery in any of the cases included in the study.

## 4. Discussion

The management of patients diagnosed with LMH is still a matter of debate, and observation is often considered a preferable option to surgery, primarily due to the relatively stable anatomical and functional characteristics of these retinal alterations, as well as the potential for unfavorable outcomes following surgical intervention [5,6,7,8]. Some authors feel that vitrectomy should be reserved for cases in which visual acuity keeps worsening or when the macular thickness progressively reduces [9,10,11]. Some studies described an increase in visual function only for ERM foveoschisis [8,12], while others reported a visual improvement following treatment of both subtypes [13,14].

Various pre-operative functional and structural biomarkers, including initial visual acuity, foveal thickness, EZ integrity, and the presence of ERP, have been investigated as potential post-operative prognostic indicators in previous studies [12,15]. Adverse prognostic markers for LMH cases undergoing surgical repair include the absence of vitreopapillary adhesion, the presence of ERP, EZ disruption, a foveal thickness of less than 100 microns, or diminished preoperative visual acuity (less than 0.2 decimal) [6,12,15]. Additionally, a recent Cochrane review evaluated only a single randomized clinical trial for surgical intervention in LMH cases due to the lack of randomized studies, highlighting the scarcity of robust evidence supporting surgical management for this condition [16,17]. A recent systematic review and meta-analysis demonstrated comparable improvements in visual acuity following PPV intervention for LMH, LMH with ERP, and ERM foveoschisis, encompassing 463 eyes across 13 studies [18]. Nevertheless, LMH and LMH with ERP subsets exhibited a heightened occurrence of post-operative full thickness macular hole.

In eyes affected by LMH, there is not a physical separation between the inner and outer retina, unlike in ERM foveoschisis, where tractional forces lead to a separation between the layers. Instead, LMH is characterized by partial loss of retinal tissue, despite the pathophysiological mechanism is unknown. One proposed theory suggests that it may manifest following anomalous posterior vitreous detachment as an abortive process of a full thickness macular hole formation [6,19]. This loss of tissue results in the accumulation of ERP in the foveal region [1]. LMH is a relatively stable pathological entity with little and slow tendency to progression and, although this stability determines a good visual prognosis for the patient, little is known about the progressive morphological changes that characterize its evolution.

In the present research, we examined the long-term outcomes of vitrectomy with ERP peeling in patients with LMH treated at our centre, evaluating both anatomical and functional results. Our findings indicated high rates of anatomical restoration and significant improvements in visual acuity, also in those eyes with low preoperative BCVA and EZ disruption. BCVA improved in all eyes, with 87.5% achieving at least 0.3 LogMAR improvement. It is noteworthy that all the patients who underwent the procedure exhibited a consistent and sustained improvement in visual acuity throughout the follow-up period of an average of 54.93 months, continuing to improve until the last follow-up. The improvement in BCVA was evident at the first postoperative month (0.24 ± 0.16 LogMAR); however, it is difficult to dissociate the visual acuity benefit deriving from cataract surgery and the visual acuity benefit deriving from membrane peel. We hypothesize that part of the improvement in BCVA recorded at the first follow-up may be due to cataract surgery, even if no patients were preoperatively affected by high grade cataract, as they were excluded from the study. Nonetheless, the additional improvement in BCVA between 1 and 6 months follow-up and the further significant improvement in BCVA between 6 months and the last follow-up by about one Snellen line indicate a progress over time and may derive from a slow, continuous retinal healing process.

Furthermore, we observed three distinct patterns of recovery: in one group of eyes (2 out of 16, 12.5%), the maximum BCVA was achieved at 1 month, for 3 eyes (18.75%), it occurred at 6 months, while the remaining 11 (68.75%) demonstrated the highest improvement at the last follow-up. These data hold significance, as although preoperative cataract was not a significant factor in operated patients, the early-month recovery could potentially be attributed to cataract extraction. The notable increase in BCVA for the majority of patients (87.5%) after the first month may indicate a meaningful progress in the retinal healing process. At the last follow-up, four patients complained of metamorphopsia during the Amsler grid test, still they subjectively reported a decrease in distortion after surgery. However, we did not use quantitative methods to analyze the entity of metamorphopsia.

In our investigation, all 16 eyes demonstrated a successful restoration of anatomical foveal contour. Additionally, among the eyes with a defect in the outer retinal layers (5 out of 16 eyes, accounting for 31.25%), there was a complete restoration of these layers. Remarkably, even eyes with initially compromised visual acuity, EZ disruption, or a thin fovea exhibited significant BVCA improvement over the course of the study. The findings are intriguing as they suggest that even patients with unfavorable preoperative prognostic indicators may experience substantial recovery if there is restoration of the external retinal layers and CFT postoperatively. The mean CFT was 211.45 microns at 1 month, increased to 248.81 microns at 6 months, and further increased to 278.37 microns at the last follow-up.

Qualitative analysis of the OCT images showed no evidence of macular edema at any follow-up examination. Therefore, the observed increase in CFT was not attributable to macular inflammation. However, it is conceivable that this increase may be associated with ongoing tissue proliferation over an extended postoperative period.

Similarly, Pertile et al. observed substantial functional and microstructural enhancements post-surgery for both ERM foveoschisis and LMH. Their findings underscore a significant reparative potential in lamellar defects of both types, thereby challenging the notion of LMH as inherently “degenerative” [20]. In concurrence with Pertile’s observations, we posit that the healing process of the LMH is gradual and continuous, as evidenced by 68.75% of the cases demonstrating maximal improvement after the 6-month follow-up. Studies with shorter follow-up durations may not capture the full extent of improvement in certain cases, potentially underestimating the restorative capacity in LMH cases.

Furthermore, the qualitative assessment of OCT images revealed the absence of ERP recurrence post-surgery, and this finding is consistent with other results from other researchers [19]. Pang et al. postulated that ERP may arise from an inner retinal defect, as the epiretinal material observed in LMH exhibits moderate reflectivity, similarly to the reflectivity observed in the middle retinal layers. They also hypothesized that Muller cells might play a role in ERP formation [4]. We may suppose that the release of traction and subsequent repair of the foveal cavity achieved after ILM peeling contribute to impede ERP, providing a plausible explanation for the absence of recurrence following successful surgery. These findings are in agreement with Pertile et al.’s hypothesis that traction underlies both ERM foveoschisis and LMH. Chehaibou et al. also endorse this theory, asserting that surgery can be advantageous for LMH patients with compromised vision [21]. Notably, ILM peeling plays a pivotal role in the procedure, leading to visual acuity improvement. ILM peeling ensures the removal of the potentially pathological posterior vitreous cortex, enhancing retinal compliance. This, in turn, relaxes the retinal surface and may contribute to the restoration of the foveal contour, enabling better anatomical and functional recovery [21,22].

Furthermore, the biological mechanisms underlying ILM peeling suggest its ability to eliminate residual cellular elements, such as Müller cell processes and other contractile components, that are critical to membrane formation and persistence. This surgical step creates a more stable retinal environment, reducing tractional forces and promoting the resolution of LMH-associated abnormalities. The dual benefit of preventing ERP and restoring the foveal contour underscores the importance of ILM peeling in LMH surgery.

In our case series, the surgical intervention for the LMHs took place at a relatively early stage, with defects in the external retinal layers present only in five eyes at baseline. It is plausible that previous studies reporting poorer outcomes may have included eyes operated at more advanced disease stages. In such cases, prolonged disease progression could have resulted in permanent impairment of the photoreceptors, thus limiting the potential for visual acuity improvement. Consequently, in alignment with recent research [20,21,23], we may postulate that the timing of surgery holds significant relevance, given that advanced disease stages may be linked to an irreversible damage to the outer retinal layers.

The risk of postoperative FTMH formation is a significant concern in the surgical management of LMH [17,21,23,24,25,26]. While the reported incidence of FTMH after surgery varies, rates up to 30% have been documented. In a recent study by Chehaibou et al., FTMH developed in 9% of operated eyes, with baseline pseudophakia and ILM peeling identified as protective factors, whereas baseline EZ disruption significantly increased the risk [21]. Modified surgical techniques, such as sparing the perihole ILM, have shown promise in reducing postoperative FTMH risk and restoring the foveal profile, although the protective effect may be limited by statistical power due to small sample sizes. In our study, no postoperative FTMH cases were observed, which may reflect the early-stage LMH in our cohort and the meticulous surgical approach, including routine ILM peeling to minimize traction on the fovea. Attention to limiting foveal stress during macular peeling remains a crucial surgical principle, especially in advanced cases where EZ and ELM disruption compromise foveal stability. Our findings underscore the importance of careful intraoperative techniques to mitigate these risks and achieve favorable anatomical and functional outcomes.

No statistically significant correlation between BCVA and CFT was identified in our study. Only a moderate positive correlation was observed between the improvement in BCVA and the increase in CFT, and this correlation weakened at the last follow-up, possibly because most patients had achieved normal or near-normal values.

The main limitations of our study were the small sample size and the retrospective collection of preoperative and early postoperative data up to 6 months after surgery. These limitations could introduce bias and potentially impact findings, such as underestimating the development of full-thickness macular hole after surgery. Another limitation of the study was its non-comparative design. Despite these limitations, our study has strength points, including the long follow-up duration and the repeatability of the surgical technique, which was performed by a single expert surgeon.

## 5. Conclusions

In conclusion, we found that vitrectomy for LMH is safe and has a good prognosis in terms of visual recovery and anatomical restoration, more evident over time, and these findings are in alignment with recent studies, sustaining the hypothesis that surgical intervention for LMH may provide a benefit in both anatomical and functional outcomes over time.

Nevertheless, further randomized prospective clinical trials with a substantial number of participants, a control group, and long term follow-up are necessary to validate our results and explore potential risk factors or complications associated with surgery.

## Figures and Tables

**Table 1:** Baseline demographic, functional, and structural characteristics of patients included in the study.

**Number of Patients (Eyes)**	14 (16)
**Sex**	
**M (%)—F (%)**	4 (28.57%)–10 (71.43%)
**Mean age (years) (SD)**	76.1 (9.9)
**Laterality**	
**RE (%)—LE (%)**	10 (62.5%)–6 (37.5%)
**Lens Status**	
**Phakic—Pseudophakic**	16 (100%)–0 (0%)
**Mean BCVA (LogMAR) (SD)**	0.46 (0.22)
**Mean CFT (micron) (SD)**	183.68 (61.73)
**ERP**	
**Yes (%)—No (%)**	13 (81.25%)–3 (18.75%)
**EZ Disruption**	
**Yes (%)—No (%)**	5 (31.25%)–9 (56.25%)
**Metamorphopsia at Amsler test**	
**Yes (%)—No (%)**	6 (37.5%)–10 (62.5%)

RE: Right eye; LE: Left eye; M: Male, F: female; BCVA: Best Corrected Visual Acuity; CFT: Central Foveal Thickness; ERP: Epiretinal Proliferation; EZ: Ellipsoid zone.

**Table 2:** Preoperative and Postoperative Best Corrected Visual Acuity in eyes affected by lamellar macular hole who underwent pars plana vitrectomy.

Time	Mean BCVA (LogMAR)	*p*-Value
**Preoperative**	0.46 (0.22)	-
**1 month**	0.24 (0.16)	<0.001 (*)
**6 months**	0.18 (0.15)	0.072 (**)
**Last follow-up**	0.09 (0.11)	<0.001 (***)

The data presented as the mean (SD). BCVA: Best Corrected Visual Acuity. (*) Comparison between preoperative BCVA and BCVA at the 1-month follow-up. (**) Comparison between BCVA at the 1-month follow-up and BCVA at 6 months. (***) Comparison between BCVA at 6 months follow-up and BCVA at the last follow-up. The statistical significance was defined as *p*-value < 0.05.

**Table 3:** Preoperative and postoperative Central Foveal Thickness in eyes affected by lamellar macular hole who underwent pars plana vitrectomy.

Time	Mean CFT (micron)	*p*-Value
**Preoperative**	183.68 (61.73)	-
**1 month**	211.45 (43.55)	<0.001 (*)
**6 months**	248.81 (48.51)	<0.001 (**)
**Last follow-up**	278.37 (45.50)	<0.001 (***)

The data presented as the mean (SD). CFT: Central Foveal Thickness. (*) Comparison between preoperative CFT and CFT at the 1-month follow-up. (**) Comparison between CFT at the 1-month follow-up vs. CFT at 6 months follow-up. (***) Comparison between CFT at 6 months follow-up vs. CFT at the last follow-up. The statistical significance was defined as *p*-value < 0.05.

## Data Availability

The data presented in this study are available on request from the corresponding author. The data (original imaging) are not publicly available due to privacy issues.
